# Early Recurrence of Thoracic Ossification of the Ligamentum Flavum After Posterior Decompression Surgery: A Case Report

**DOI:** 10.7759/cureus.59429

**Published:** 2024-05-01

**Authors:** Sho Asanuma, Kousei Miura, Toru Funayama, Masao Koda, Masashi Yamazaki

**Affiliations:** 1 Department of Orthopaedic Surgery, Institute of Medicine, University of Tsukuba, Tsukuba, JPN

**Keywords:** case report, mechanical stress, recurrence, thoracolumbar junction, ossification of the ligamentum flavum

## Abstract

Thoracic ossification of the ligamentum flavum (OLF) is known to result in spinal canal stenosis and myelopathy. It is typically treated through decompressive laminectomy and resection of the ossified ligament, which is known to improve neurological deficits. However, the recurrence of OLF post-surgery remains a relatively undocumented and complex issue. The present report describes the case of a 58-year-old male patient who had obesity (BMI 34), diabetes mellitus, and Basedow's disease. The patient presented with bilateral lower limb paresthesia and associated gait impairment, resulting in an urgent hospital admission. Imaging diagnostics identified extensive thoracic ossification of the posterior longitudinal ligament and OLF, both of which resulted in significant spinal cord compression. He underwent posterior decompression with instrumented fusion from T1 to T9 and additional laminectomy and OLF resection at T10/11. Despite an initial improvement in the postoperative period, the patient developed an epidural hematoma one week following surgery, causing significant paralysis of the lower limbs. This complication was promptly addressed with hematoma removal surgery. Six months after the initial procedure, his walking function improved significantly, but eight months after surgery, he experienced a sudden regression in motor functions due to the recurrence of OLF at T10/11, necessitating an additional posterior instrumented fusion surgery. Subsequent to the additional surgical procedure, the patient experienced an amelioration in paralysis, enabling him to ambulate with the aid of a cane. The recurrence of thoracic OLF after decompression surgery is a significant concern, especially in cases where decompression without instrumented fusion is performed. When determining the surgical procedure for thoracic OLF in cases with extensive ossification of the spinal ligaments, it is crucial to consider the degree of spontaneous fusion and mobility of the spinal segments, as demonstrated in the present case. The concentration of mechanical stress due to fusion at adjacent segments and intervertebral mobility at the thoracolumbar junction may increase the risk of OLF recurrence and should be carefully assessed preoperatively, even though posterior decompression surgery is typically considered a sufficient option for thoracic OLF.

## Introduction

Thoracic ossification of the ligamentum flavum (OLF) can potentially cause spinal canal stenosis and myelopathy [1]. The literature characterizes its development as an intricate combination of genetic, mechanical, and metabolic factors resulting in progressive deterioration [1]. One commonly used method for treating progressive myelopathy because of thoracic OLF is surgery, specifically decompressive laminectomy and resection of the ossified ligament. These procedures are reported to be effective in improving neurological deficits [1-3].

Nevertheless, the occurrence of OLF after surgery continues to be a mysterious and poorly documented phenomenon. In this report, we describe a case of a middle-aged man with extensive ossification of spinal ligaments who required additional surgery due to an early postoperative recurrence of OLF.

## Case presentation

The patient, a 58-year-old male with obesity (106 kg, BMI 34), presented with bilateral lower limb paresthesia and ambulatory difficulties four months prior to the initial surgery. He had a medical history of diabetes mellitus and Basedow's disease, both of which were treated with medication. He experienced a precipitous exacerbation of his walking impairment, necessitating an emergency hospital admission.

At the initial examination in our institution, the patient exhibited pervasive sensory deficits below the inguinal region, coupled with motor impairment in both lower limbs (manual muscle testing (MMT) score 3-4/5), compromising his ability to stand. Urinary incontinence and residual urine, which are signs of bladder and bowel dysfunction, made his condition complicated. Hyperactive deep tendon reflexes were observed in both lower limbs. The Japanese Orthopedic Association's (JOA) scoring system for cervical myelopathy, excluding scores for the upper extremities, was 4 points (full score: 11 points).

**Figure 1:**
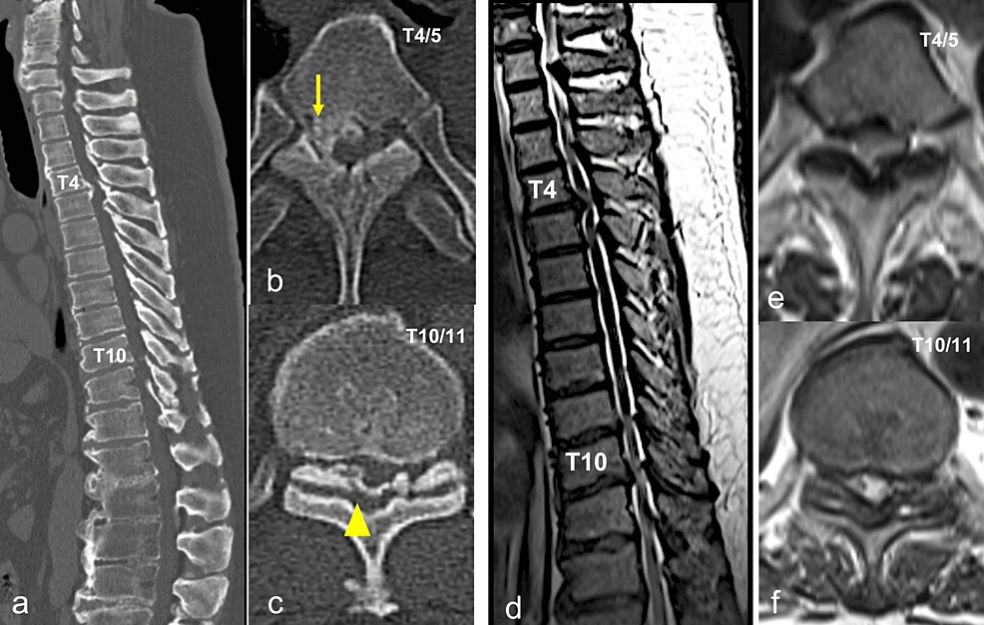
Preoperative images Sagittal reconstruction (a) and axial (b, c) CT show OPLL at T4/5 (yellow arrow) and OLF at T10/11 (yellow arrowhead). Sagittal (d) and axial (e, f) T2-weighed images show significant spinal cord compression at these levels. OPLL: ossification of the posterior longitudinal ligament, OLF: ossification of the ligamentum flavum

Imaging assessments showed the presence of ossification of the posterior longitudinal ligament (OPLL) at the T4/5 level and OLF at the T4/5, T5/6, and T10/11 levels on plain CT scans. Additionally, MRI demonstrated severe compression of the spinal cord at these levels (Figure 1).

**Figure 2:**
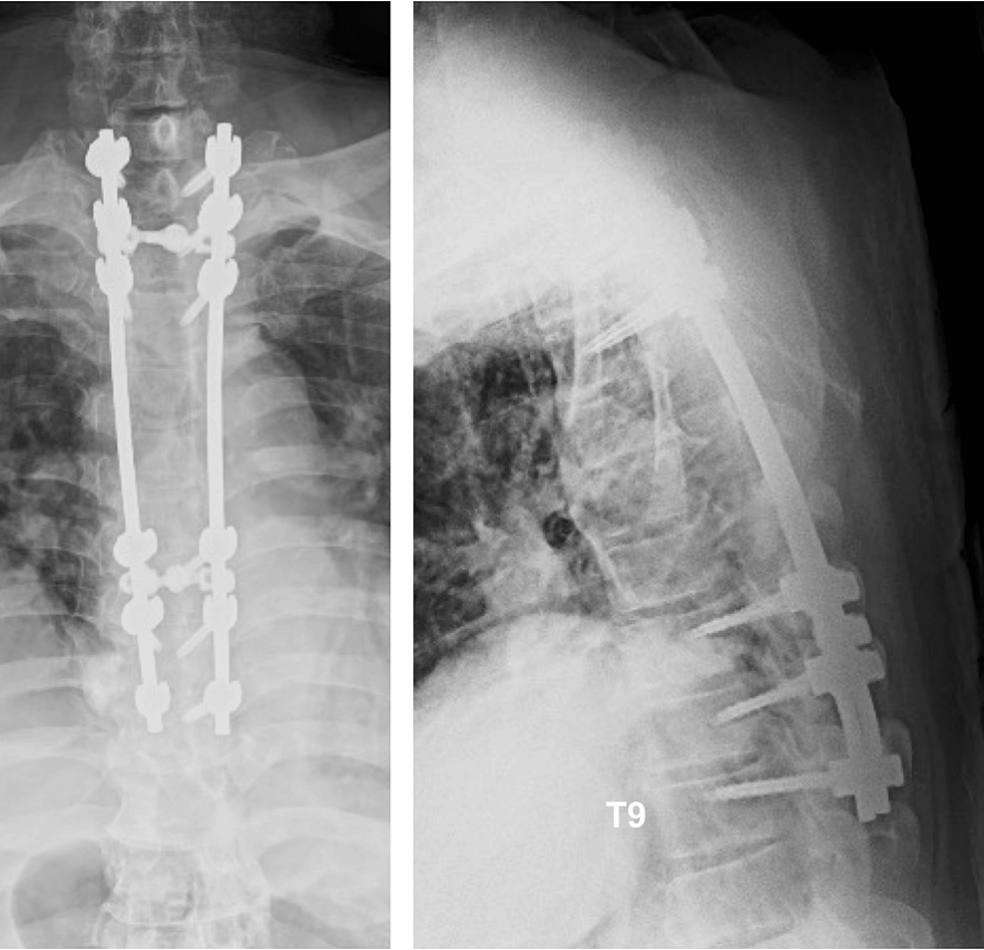
Radiographs after initial surgery Radiographs after posterior decompression and fixation at T1–9, along with laminectomy and OLF resection without instrumented fusion at T10–11. OLF: ossification of the ligamentum flavum

His diagnosis was thoracic myelopathy, which developed from extensive thoracic OPLL and OLF. A surgical procedure including posterior decompression with instrumented fusion was carried out at T1-T9, along with laminectomy and OLF resection without instrumented fusion at T10-11 (Figure 2).

The surgery was successfully completed without any intraoperative complications. However, a week after surgery, an epidural hematoma caused significant lower limb paralysis (MMT score 1-2/5). Epidural hematomas pooled extensively between T3 and T6. This was quickly treated with hematoma removal surgery, which improved the functionality of the lower limbs.

**Figure 3:**
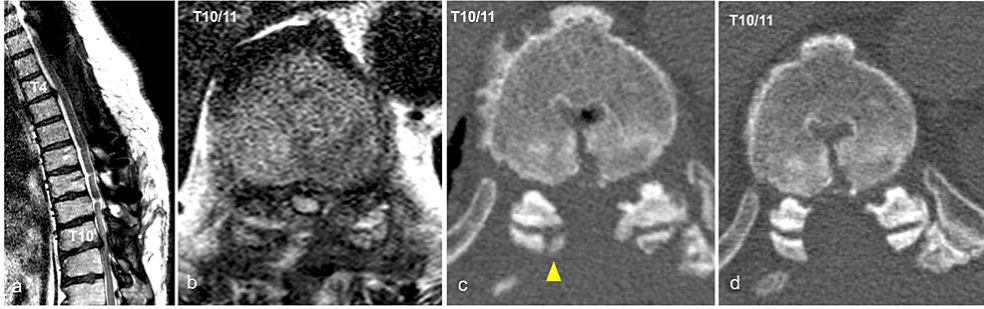
Images before revision surgery Sagittal (a) and axial (b) T2-weighted MRI images show severe spinal cord compression at T10/11. Axial CT before revision surgery (c) shows a recurrence of OLF at T10/11 (yellow arrowhead) compared to axial CT immediately after the initial surgery (d). MRI: magnetic resonance imaging, CT: computed tomography, OLF: ossification of the ligamentum flavum

Six months after the initial surgery, this patient got better enough that he could walk with a cane, and his JOA score went up to 6 points. Nevertheless, eight months after the initial surgery, the motor weakness of his lower limb deteriorated unexpectedly, resulting in an impairment of his ability to stand. Right iliopsoas and quadriceps motor weakness (MMT score 2/5) and a decreased JOA score of 3 points were discovered upon emergency transfer to our hospital. Plain CT scans and MRI revealed spinal cord compression due to an OLF recurrence at the T10/11 (Figure 3). Thus, bony canal stenosis due to OLF recurrence, intervertebral instability, and disc bulging were considered to have led to myelopathy.

**Figure 4:**
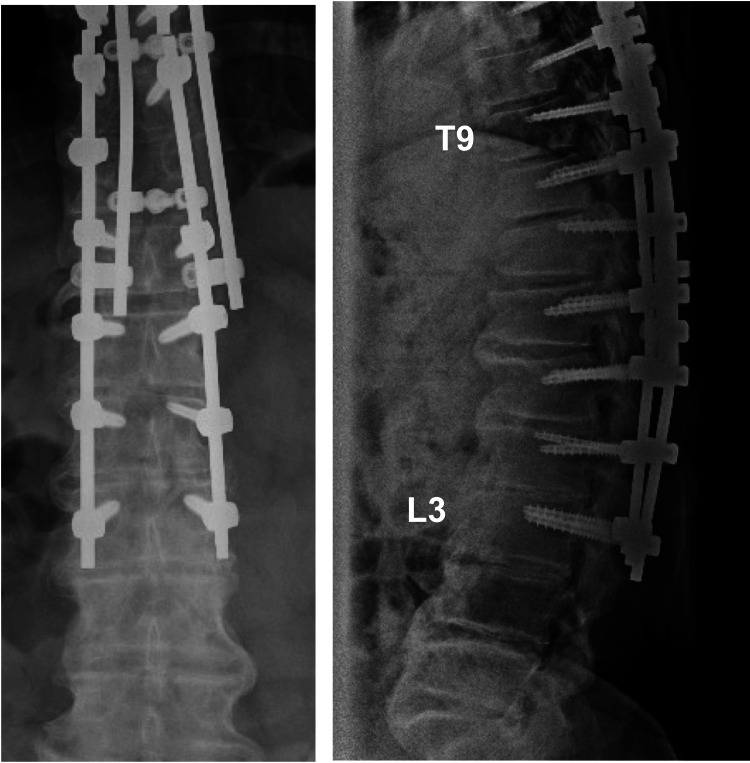
Radiographs after revision surgery Radiographs after removing the recurring OLF at the T10/11 and extending the posterior fixation to the L3. OLF: ossification of the ligamentum flavum

A further surgical procedure was performed to remove the recurring OLF at the T10/11 and to extend the posterior fixation to the L3 (Figure 4). Spinal cord compression due to bony stenosis of the ossificated ligamentum flavum looked apparent.

After the additional surgery, his paralysis got better, allowing him to walk with the use of a cane. His ultimate JOA score was 6 points.

## Discussion

In this case, posterior decompression with instrumented fusion from T1 to T9 was performed on the OPLL and OLF in the middle thoracic spine, and laminectomy and OLF resection without instrumented fusion were initially performed on the OLF of T10/11 at the thoracolumbar junction. In general, posterior decompression with the removal of thoracic OLF has been found to be a successful surgical treatment for thoracic OLF [2,3]. Posterior decompression, which includes medial facet joint decompression, does not cause instability. Therefore, the use of posterior fixation is not considered necessary [1]. Therefore, in the present case as well, posterior decompression alone was initially performed at T10/11, where the spinal cord had been compressed solely by OLF, resulting in temporary improvement of thoracic myelopathy.

**Figure 5:**
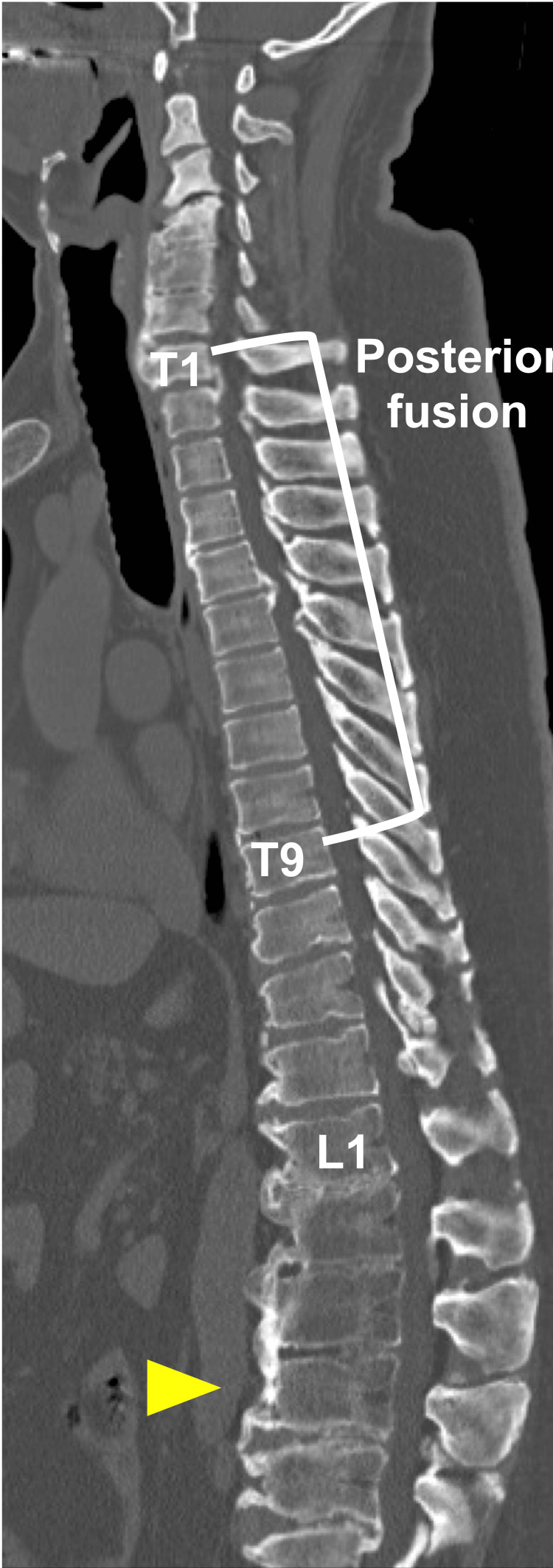
Preoperative whole spine sagittal reconstruction CT image There was a spontaneous fusion of the anterior longitudinal ligament ossification between L1 and S1 (yellow arrowhead). Additionally, the first surgery added posterior instrumented fusion from T1 to T9, limiting the intervertebral mobility of the thoracolumbar spine to the thoracolumbar junction only. CT: computed tomography

Nevertheless, the OLF that was surgically removed at T10/11 recurred shortly after the initial surgery. OLF recurrence and intervertebral instability led to the need for additional surgery due to the recurrence of myelopathy. There have been several case reports of thoracic OLF recurrence after decompression alone without fixation. Ando et al. performed a laminectomy at the T1-11 and posterior fixation at the T6-9 in a case with extensive thoracic OPLL and OLF. Postoperatively, the patient developed recurrent OLF at T10/11, which was also affected by spontaneous fusion of the ossification of the anterior longitudinal ligament (OALL) at T12-L1 [4]. Kanno et al. documented two cases of OLF recurrence following decompression surgery. Interestingly, both cases occurred at the thoracolumbar junction and involved the fusion of the facet joint caudal to the lesion [5]. It is fascinating to observe that all cases, including ours, occurred at the thoracolumbar junction. In addition, Funayama et al. reported a case of thoracic myelopathy due to a progression of OLF at T9/10 after C7-T8 posterior decompression with instrumented fusion in a patient with extensive thoracic OPLL and OLF. This case also had a spontaneous fusion of the OALL at T7-9, suggesting that the OLF enlarged as a result of high mechanical stress at T9/10 [6]. Both reports showed that additional posterior decompression with instrumented fusion led to improvements in myelopathy, as in our case. These reports indicated that spontaneous fusion at adjacent levels and the increased mobility of the thoracolumbar junction could have led to the re-extension of the OLF, possibly due to the concentration of mechanical stress on the lesion site [4-6]. Also, in the present case, posterior instrumented fusion was carried out at T1-T9 at the initial surgery, where spontaneous fusion of the OALL at L1-S1 was present (Figure 5).

Therefore, it is speculated that the mechanical stress concentration at the thoracolumbar junction might have contributed to the early postoperative recurrence of OLF after the resection. Furthermore, in addition to OLF recurrence, intervertebral instability and disc bulging could have contributed to myelopathy deterioration.

It may be worth considering whether decompression surgery alone, without instrumented fusion, is sufficient for OLF in the thoracolumbar junction, while there have been some positive reports regarding the effectiveness of decompressive instrumented fusion for thoracic OLF [7,8]. The available evidence is insufficient for making clear judgments. It remains controversial whether to perform posterior instrumented fusion in addition to decompression for thoracic OLF in the initial treatment [2,9]. However, based on the present case, it is important to consider the extent of spontaneous fusion and mobility of the spinal segments when determining the surgical procedure for thoracic OLF in cases with extensive ossification of the spinal ligaments.

## Conclusions

Although posterior decompression surgery alone is generally considered a sufficient option for thoracic OLF, the concentration of mechanical stress due to fusion at adjacent segments and intervertebral mobility at the thoracolumbar junction may pose a risk of OLF recurrence and should be thoroughly evaluated preoperatively. Particular attention should be given to cases involving extensive ossification of the spinal ligaments, as shown in this case. It is important to consider posterior decompression with instrumented fusion in cases where OLF recurrence is a concern.
